# Machine learning insights on the effectiveness of non-pharmaceutical interventions against COVID-19 in Nigeria

**DOI:** 10.1093/inthealth/ihae065

**Published:** 2025-01-09

**Authors:** Kolapo M Oyebola, Funmilayo C Ligali, Afolabi J Owoloye, Blessing D Erinwusi, Adesola Z Musa, Oluwagbemiga O Aina, Babatunde L Salako

**Affiliations:** Centre for Genomic Research in Biomedicine, Mountain Top University, Ibafo, Nigeria; Nigerian Institute of Medical Research, Lagos, Nigeria; Centre for Genomic Research in Biomedicine, Mountain Top University, Ibafo, Nigeria; Nigerian Institute of Medical Research, Lagos, Nigeria; Centre for Genomic Research in Biomedicine, Mountain Top University, Ibafo, Nigeria; Nigerian Institute of Medical Research, Lagos, Nigeria; Centre for Genomic Research in Biomedicine, Mountain Top University, Ibafo, Nigeria; Nigerian Institute of Medical Research, Lagos, Nigeria; Nigerian Institute of Medical Research, Lagos, Nigeria; Nigerian Institute of Medical Research, Lagos, Nigeria

**Keywords:** clustering algorithms, COVID-19, epidemic responses, non-pharmaceutical interventions, outbreak, regression analysis

## Abstract

**Background:**

The lack of effective pharmacological measures during the early phase of the COVID-19 pandemic prompted the implementation of non-pharmaceutical interventions (NPIs) as initial mitigation strategies. The impact of these NPIs on COVID-19 in Nigeria is not well-documented. This study sought to assess the effectiveness of NPIs to support future epidemic responses.

**Methods:**

Daily COVID-19 cases and deaths were analysed using smoothed variables to identify transmission trends. Regression analysis and clustering algorithms were applied to evaluate the impact of each NPI.

**Results:**

Multiple transmission peaks were reported, with the highest smoothed daily new cases (approximately 1790) observed around 29 December 2021 and smoothed daily new deaths (approximately 23) peaking around 8 September 2021. NPIs such as public transport (coefficient value −166.56, p=0.01) and workplace closures (coefficient value −150.06, p=0.01) strongly correlated with decreased case numbers. This finding highlights the importance of mobility control and non-essential workplace management in slowing infection transmission during an outbreak. Public transport restrictions (coefficient value −2.43, p<0.001) also had a direct effect on death reduction.

**Conclusions:**

Public transport restrictions and workplace closures correlated with reductions in the number of cases and deaths. These findings can guide future pandemic responses to enhance favourable public health outcomes.

## Introduction

The global health crisis caused by the emergence of severe acute respiratory syndrome coronavirus 2 (SARS-CoV-2) necessitated a global public health response.^1,2^ As vaccines and effective therapeutic options were largely unavailable during the early stages of the pandemic, public health stakeholders implemented non-pharmaceutical interventions (NPIs) as primary response strategies to contain viral transmission and safeguard public health.^3–5^ Given the widespread use of NPIs during the early stages of the pandemic, it is essential to evaluate their effectiveness to better inform future public health strategies. This evaluation is particularly important for Nigeria, where the impact of NPIs on public health needs to be understood in the context of local challenges and health system capacities. Evaluating the benefits of NPIs will provide valuable insights into optimizing public health responses to enhance preparedness for emerging or re-emerging infectious diseases in the region.

Randomized controlled trials (RCTs) are often considered the gold standard for evaluating the effectiveness of interventions.^6,7^ However, conducting RCTs during a pandemic poses significant ethical and logistical challenges. Specifically, randomizing populations to different intervention arms during an ongoing outbreak could portend life-threatening implications.^8^

Mathematical modelling of disease transmission dynamics has traditionally been used to investigate the impact of NPIs on the spread of infectious diseases.^9–12^ These models rely on a set of assumptions and parameters that describe the interactions between susceptible, exposed, infectious and recovered individuals within a population.^13,14^ While this approach is useful, the accuracy of the model depends on the validity of underlying assumptions, which may not always hold true during a rapidly evolving pandemic.^15,16^ Factors such as changes in virus transmissibility, public adherence to NPIs and the introduction of new interventions can significantly affect the accuracy of the model.^17–19^ These challenges highlight the need for alternative approaches to evaluate NPIs. Machine learning (ML) algorithms offer a promising alternative for evaluating NPIs. ML tools can analyse vast amounts of data to identify patterns that may not be apparent through traditional statistical methods.^20–22^ This approach is particularly valuable during a pandemic, where real-time data on case numbers, positivity rates and deaths can be continuously updated and analysed.

In this study, we explored clustering algorithms and regression analysis for a retrospective evaluation of NPIs against COVID-19 in Nigeria. We analysed temporal data on COVID-19 cases and deaths. We also assessed the effectiveness of different NPIs in containing the spread of the virus and mitigating their impact on overall public health outcomes.

## Methods

### Data collection

De-identified COVID-19 data were sourced from Our World in Data,^23^ an open-access database (https://ourworldindata.org/coronavirus). The granular dataset included epidemiological (e.g. confirmed cases and deaths), demographic, healthcare and intervention information.

### Description of NPIs

The NPIs investigated include face coverings, school closures, workplace closures, public transport cancellation, cancellation of public events, stay home requirements, contact tracing, testing policy and vaccination policy (Supplementary Table S1). The use of facial covering was categorized into five levels of policy implementation: no policy (0); recommended (1); required in specific shared or public spaces outside the home where others were present, or in situations where social distancing was not feasible (2); required in all shared or public spaces outside the home where others were present or in all situations where social distancing was not feasible (3) and required outside the home at all times, irrespective of location or presence of others (4). Regarding school and workplace closures, the following categories were specified: no data available (NaN), no measures implemented (0), recommendations in place (1), required closures at certain levels (2) and required closures at all levels (3). Public event cancellations were categorized as no data available (NaN), no measures implemented (0), recommended cancellations (1) and required cancellations {2}. Stay home policies were grouped into four categories: no measures implemented (NaN); recommended stay home advisories (0); required stay home orders with exceptions for essential activities like exercise, shopping and necessary trips (1) and required stay home orders with minimal exceptions, such as leaving the house only once every few days or limiting outings to one person at a time (2). Categories of public transport closures included no data available (NaN), no measures implemented (0), recommended closures or reduced service (1) and required closures or significant restrictions on usage (2). Lastly, vaccination policies had six categories: no availability (0); availability for one of the following groups: key workers, clinically vulnerable groups, elderly groups (1); availability for two of the following groups: key workers, clinically vulnerable groups, elderly groups (2); availability for all three groups: key workers, clinically vulnerable groups, elderly groups (3); availability for all three groups plus partial additional availability for select broad groups or ages (4); and universal availability (5). This was solely a vaccination availability policy and did not reflect the actual vaccination coverage, which was not the focus of this current study.

### Feature selection and engineering

Data cleaning, exploratory analysis and feature engineering were performed in Google Colab with Python 3.10 (https://github.com/oyebolakolapo/Retrospective-Evaluation-of-Non-Pharmaceutical-Interventions-against-COVID-19-in-Nigeria.git). Missing values (NaN) were expunged from the analysis (Supplementary Figure S1). We extracted features such as intervention measures (e.g. testing policy, contact tracing, vaccination policy, face coverings, stay home restrictions, school and workplace closures, embargo on public events and public transportation), sociodemographic factors (e.g. population density, human development index, gross domestic product per capita, extreme poverty rate) and healthcare capacity (e.g. hospital beds, intensive care unit capacity, handwashing facilities) from the dataset. A p-value ≤0.05 was considered statistically significant.

### Correlation analysis

To identify the NPIs associated with a reduction in COVID-19 spread, we computed the Pearson correlation coefficients between individual intervention measures and key epidemiological metrics, including new cases and deaths. Interventions demonstrating strong negative correlations with these outcomes were considered to have a greater impact on controlling the transmission of the virus. To facilitate interpretation of the correlation results, we utilized scatter plots and heat maps. These graphical representations enabled us to identify patterns and trends in the data and assess the relative effectiveness of various intervention strategies.

### K-means clustering and silhouette scoring

K-means clustering offers advantages in grouping data points with similar characteristics, making it a powerful tool for identifying patterns in intervention measures. As such, we implemented K-means clustering algorithms to group data points or intervention measures with similar characteristics to enable the identification of potential patterns in implementation effectiveness or impact. To achieve this, we determined the optimal number of clusters (K) using the elbow method.^24^ The optimal number of clusters is at the ‘elbow’ of the graph, where the inertia would decrease slowly if the number of clusters was increased. In addition, we employed silhouette scoring as a metric to assess the coherence and separation of clusters generated through these algorithms.^25^ Silhouette scores were calculated for each data point to provide insights into the quality of clustering by measuring the similarity within clusters compared with neighbouring clusters. This evaluation process allowed us to identify the optimal number of clusters to ensure robustness of the analysis. After identifying the optimal number of clusters using silhouette scoring, we applied principal component analysis (PCA) to reduce the dimensionality of the data and visualize the cluster distributions in a two-dimensional (2D) space. PCA was used post-clustering to provide a clear visual representation of how interventions grouped within each cluster, which made it easier to interpret relationships and overlaps between intervention policies.

### Regression model

The performance of the regression model in predicting the number of COVID-19 cases and deaths following the implementation of NPIs was evaluated. Two scatter plots were generated to visualize the relationship between predicted and actual values, one for cases and one for deaths. Moreover, a regression line was fitted to the data to illustrate the overall trend of predictions compared with actual values. The predictive accuracy of the model was then assessed by comparing the scatter plots to diagonal dashed lines. The determination of predictive accuracy was facilitated by calculating the R^2^ values, as presented in Supplementary Table S2.

## Results

### Trend analysis and transmission peaks

**Figure 1.**
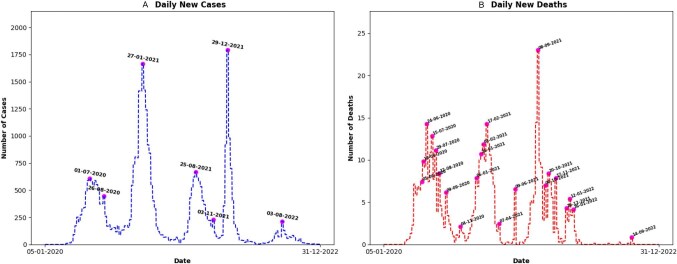
Trend analysis of daily new COVID-19 **(A)** cases (smoothed) and **(B)** deaths (smoothed) in Nigeria from January 2020 to December 2022. The coinciding peak dates are indicated on the curve. DD-MM-YYYY date format was used in the plots.

We determined the number of daily new COVID-19 cases and deaths from 5 January 2020 to 31 December 2022 to identify transmission trends, peaks as well as troughs. We adopted the mathematically adjusted (smoothed) variables of daily new cases and deaths in our analysis to control for daily fluctuations and provide a clear picture of underlying trends. The first reported case in the country was on 1 March 2020, peaking on 1 July 2020 with approximately 610 new smoothed cases (Figure 1A). Short intervals between peaks of new cases were observed during the early months of the outbreak, particularly from 1 July 2020 to 27 January 2021, after which a longer trough interval was observed until 25 August 2021. However, the peak intervals narrowed shortly afterwards until 29 December 2021, when the number of new cases reached the maximum peak before plummeting. The report of new COVID-19-related deaths followed a somewhat different pattern. The first reported COVID-19-linked death in the country was on 29 March 2020, peaking on 6 May 2020, with approximately seven new smoothed cases (Figure 1B). There were a couple more peaks within short intervals after which the maximum peak of new daily deaths was recorded on 8 September 2021. The last documented daily new death (smoothed) was reported on 18 September 2022.

### Correlation analysis

**Figure 2.**
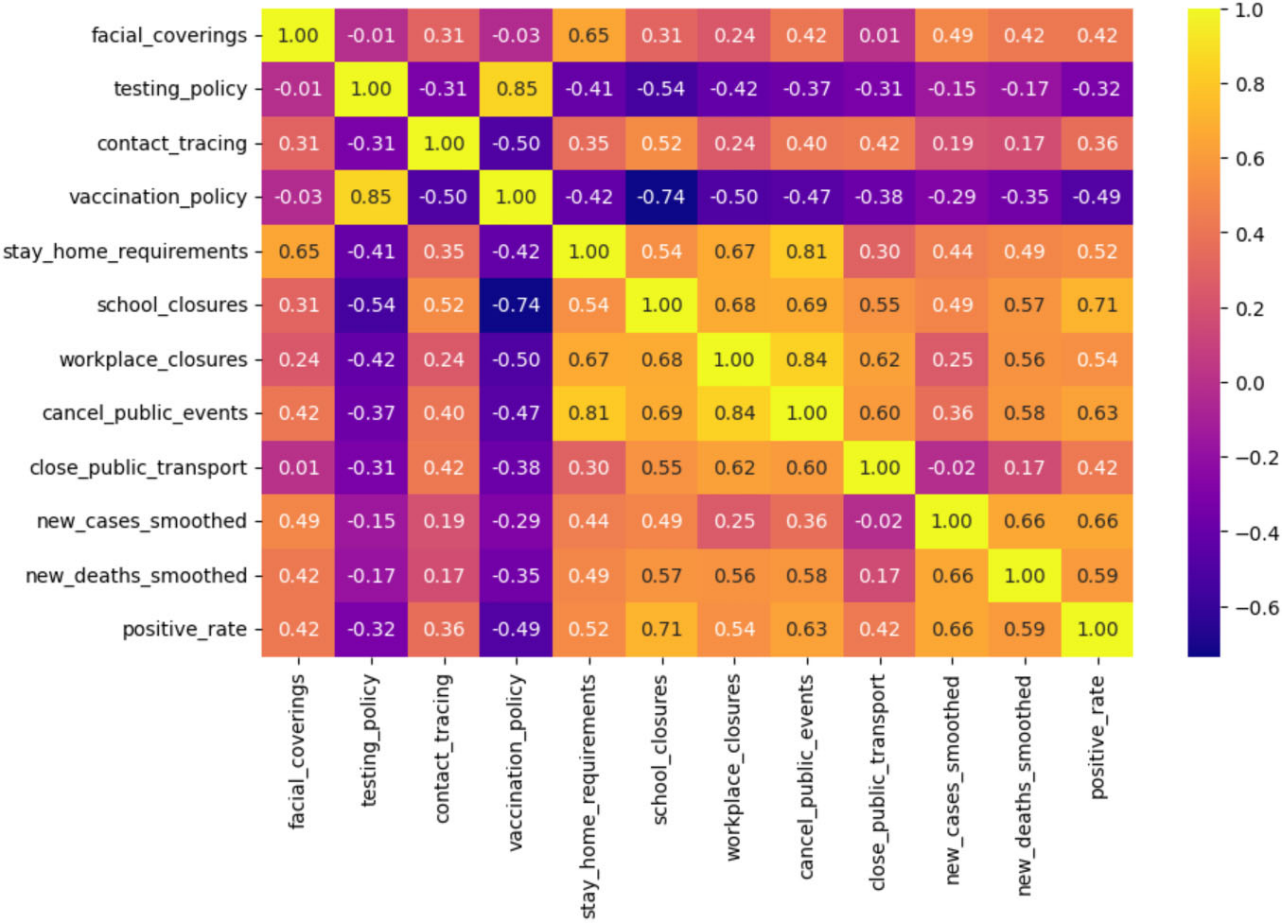
Correlation matrix of relationships between non-pharmaceutical COVID-19 interventions and outcomes. The values ranged from −1 (perfectly negative correlation) to 1 (perfectly positive correlation), with 0 indicating no linear association. Strong positive correlations (yellow squares) indicate a direct correlation between variables while strong negative correlations (purple squares) indicate an inverse trend.

**Figure 3.**
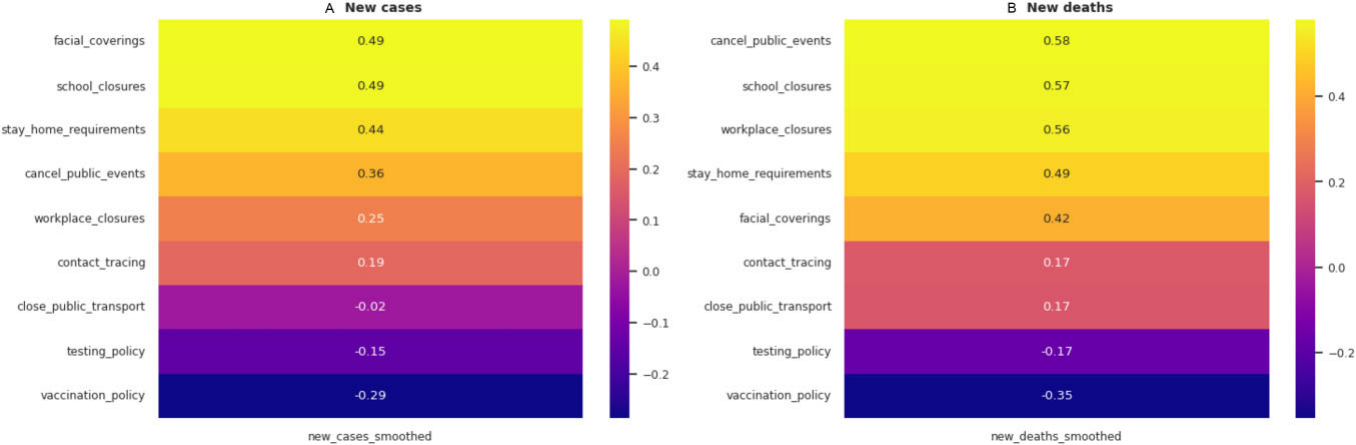
Correlation between NPIs and new COVID-19 **(A)** cases and **(B)** deaths. High correlation values suggest lower new COVID-19 cases and deaths with NPIs.

In Figures 2 and 3, we show how different COVID-19 interventions related to each other and how they possibly influenced new cases and deaths. High correlations were observed between certain intervention measures (Figure 2). For instance, testing and vaccination policies (0.85), cancellation of public events and workplace closures (0.84), cancellation of public events and stay home requirements (0.81) were social isolation strategies that were likely implemented together or had similar effects on the epidemiology of the virus in the country. Cancellation of public events and school closures (0.69), workplace and school closures (0.68), workplace closures and stay home requirements (0.67) and school closures and public transport cancellation (0.55) were also moderately correlated.

To determine the interventions that were most effective in reducing the spread of the virus, we visualized their correlation with the number of new COVID-19 cases (Figure 3A). Facial covering and school closures showed moderate positive correlation (0.49) with fewer new cases, followed by stay home requirements (0.46), while cancellation of public events (0.36) was weakly correlated. Similarly, cancellation of public events (0.58) showed the highest positive correlation with the number of COVID-19-related deaths (Figure 3B). This was followed by school (0.57) and workplace (0.56) closures, respectively. Conversely, testing and vaccination policies were weakly correlated with a reduction in new cases (−0.15 and −0.29, respectively), deaths (−0.17 and −0.35, respectively) and positive rates (−0.32 and −0.49, respectively).

### K-means clustering

**Figure 4.**
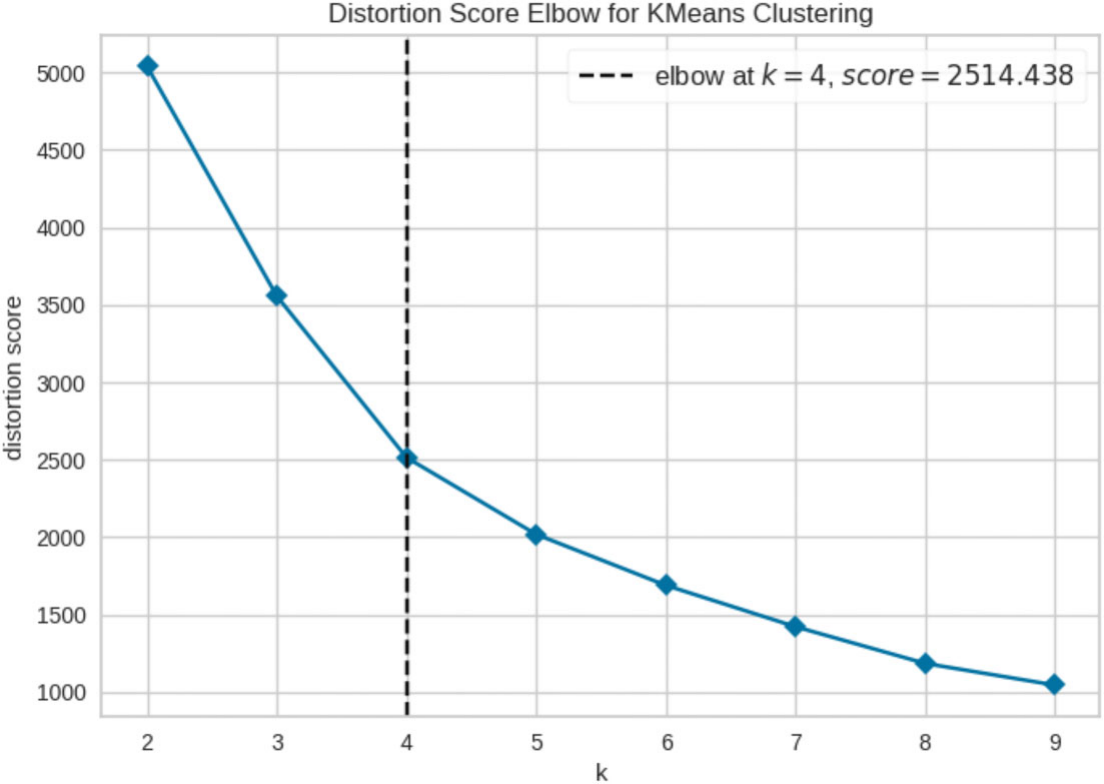
Elbow plot to determine the optimal number of clusters of NPIs in the K-means clustering algorithm. The graph shows the within-cluster sum of squares (WCSS) values on the y-axis corresponding to the different values of K (on the x-axis). The optimal K value is the point at which the graph forms an elbow.

To determine intervention measures with similar characteristics and identify potential patterns in implementation effectiveness, we implemented a K-means clustering algorithm. The elbow plot in Figure 4 displayed inertia (within-cluster sum of squares) as a function of the number of clusters (K). As the number of clusters increased, the inertia decreased because each data point could be assigned to a cluster more closely. However, at some point, the rate of decrease slowed, forming an elbow in the plot. This point indicated the optimal number of clusters and represented a balance between minimizing inertia (making the clusters internally coherent) and avoiding overfitting (i.e. creating too many clusters that might not generalize well to new data). In this case, the elbow point suggested an optimal choice of four clusters (Figure 4).

### Silhouette scoring and PCA

**Figure 5.**
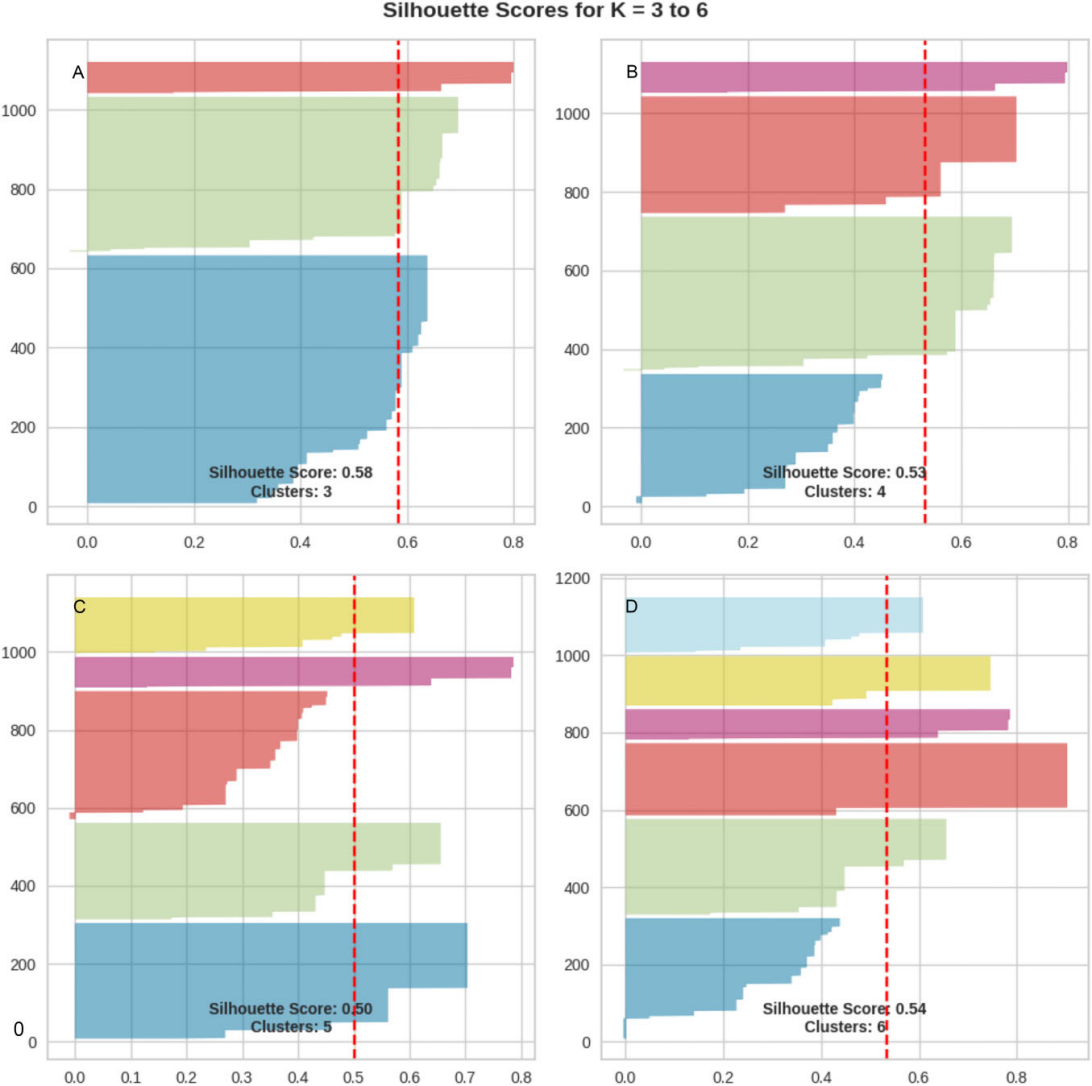
Silhouette scores of cluster numbers. Each individual object is represented on the y-axis and the red dashed line indicates the silhouette coefficient for that sample. A higher silhouette coefficient indicates better clustering quality for the corresponding sample. The x-axis represents the silhouette score.

**Figure 6.**
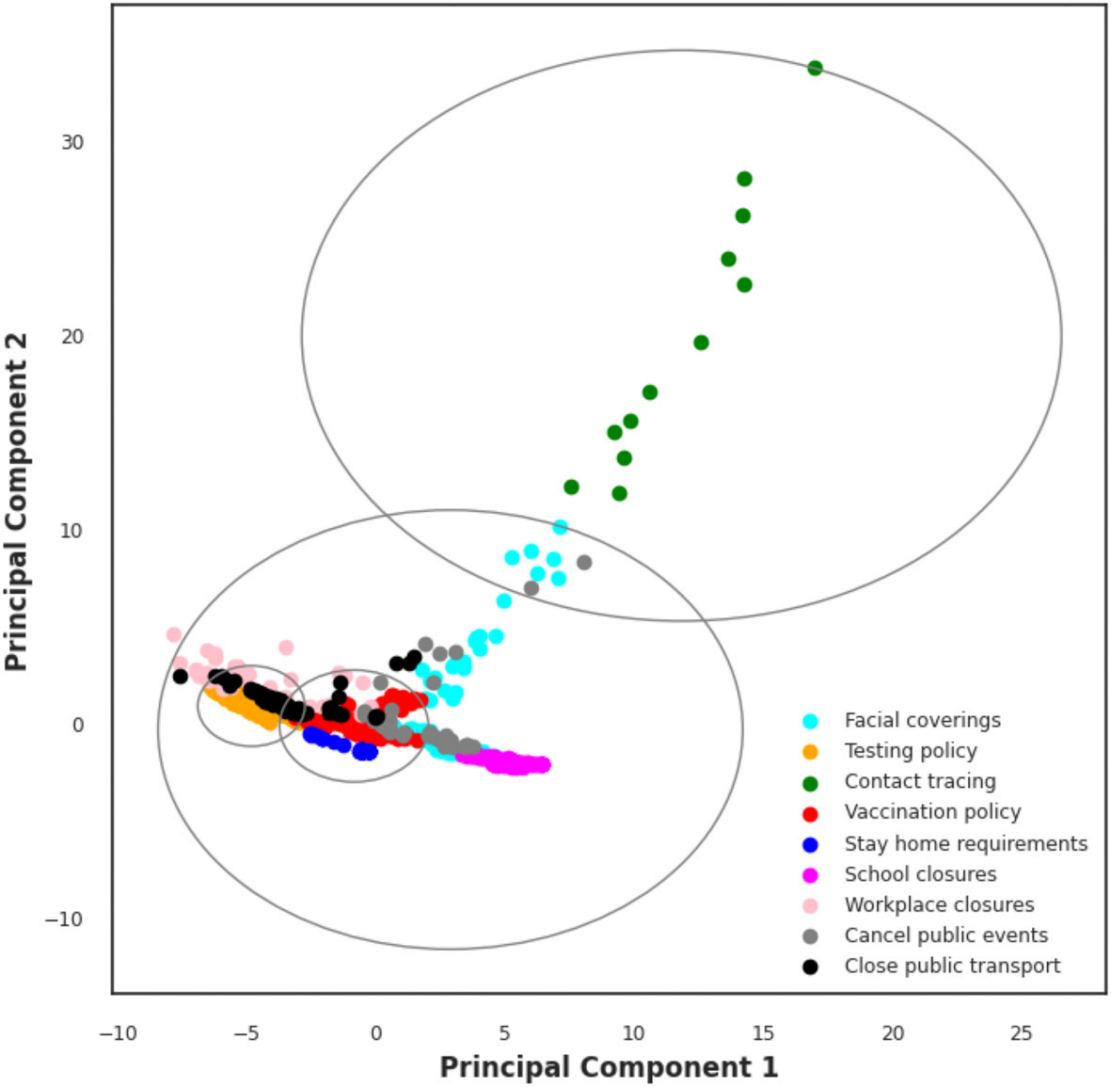
The 2D PCA of intervention data points and boundary circles to delineate the main clusters. The grey circles represent the boundary circles for the main clusters. These circles are drawn around the centroids of the main clusters, with a radius equal to the maximum distance from any point within the cluster to its centroid. The circles serve as a visual representation of the extent or spread of each main cluster.

In real-world datasets, there is often no clear elbow inflection point to identify the right ‘K’ using the elbow method, as was the case in this study. To circumvent this, we calculated the silhouette scores of possible clusters (K=3–6). The silhouette score was maximum (0.58) for K=3 (Figure 6), but that was not the final index for optimal K determination. For a given K, all the clusters were expected to have a silhouette score (represented by the red dotted line in Figure 6) greater than the average score of the sample set represented. Only cluster 3 followed this assumption, so K=3 was selected as our optimal cluster. The 2D data points of NPIs after dimensionality reduction were represented using PCA. Each point represents an intervention, with points grouped to the same cluster suggesting similarities or overlap between intervention policies (Figure 5). Clusters that were more spread out represented interventions with diverse or conflicting responses across selected features. For instance, data points for contact tracing seemed to form a distinct cluster (Figure 6; Supplementary Figure S2).

### Regression model

**Figure 7.**
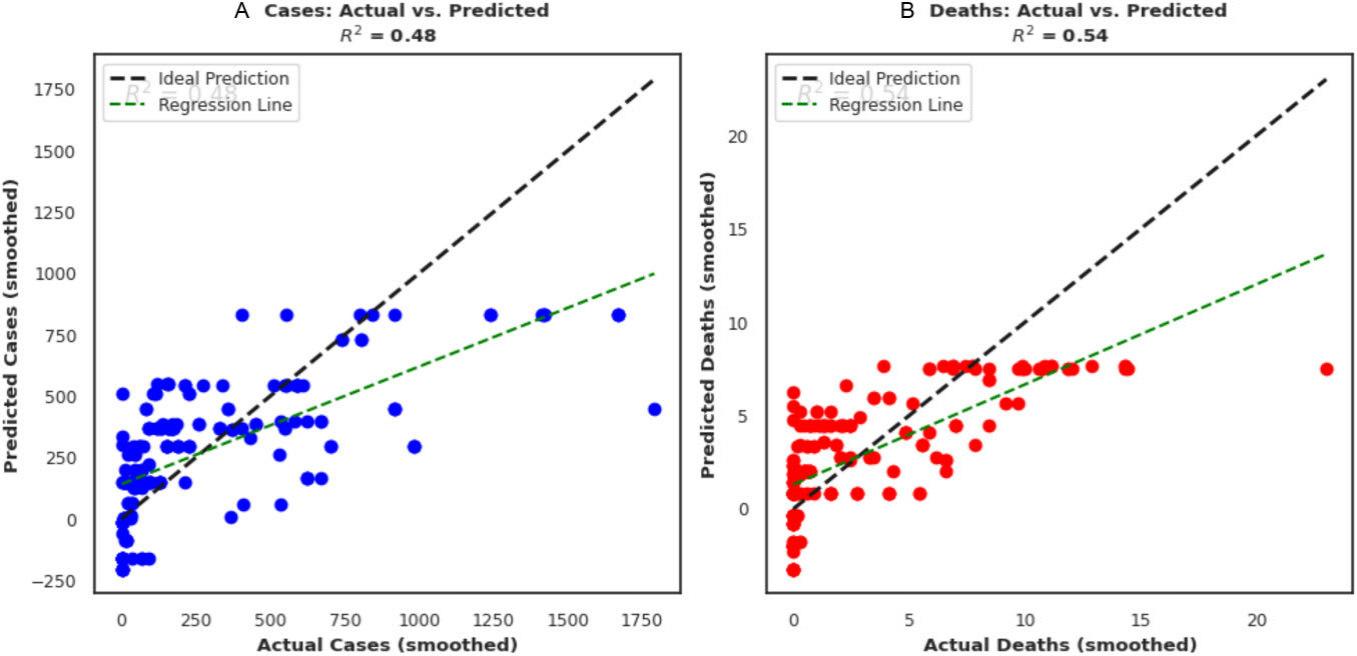
Regression model of predicted and actual new **(A)** cases and **(B)** deaths following non-pharmaceutical COVID-19 interventions. Each plot shows actual values (x-axis) versus predicted values (y-axis) for data points in the testing set. The diagonal dashed line illustrates perfect predictions, where the predicted and actual numbers of cases matched. The green dashed lines in the scatter plots represent the overall trend between actual and predicted values. A regression line closely following the diagonal line suggests a strong linear relationship between the model's predictions and the actual data. R^2^ values range from 0 to 1, with higher values indicating a better fit between the model's predictions and the actual data.

**Figure 8.**
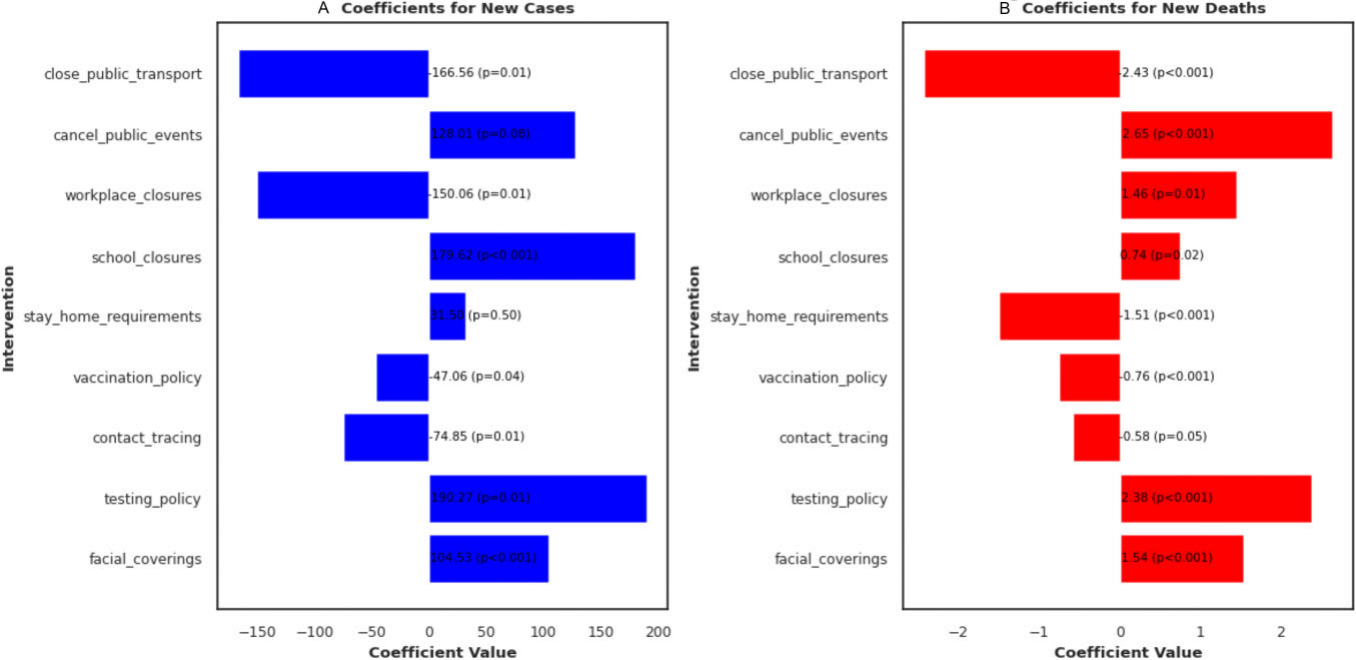
Coefficients of the effect of NPIs on new COVID-19 **(A)** cases and **(B)** deaths. Negative coefficients suggest a decrease in predicted cases/deaths with an increase in the intervention and vice versa. The absolute value of the coefficient reflects the strength of the effect, with larger values indicating a stronger impact on the prediction.

We evaluated the regression model's performance for predicting the number of cases and deaths following NPI implementation. Two scatter plots were generated, one for cases and one for deaths (Figure 7). In addition, a regression line was fitted to the data, depicted by the green dashed line indicating the overall trend of predictions compared with actual values. By comparing the scatter plots with the diagonal dashed lines, we assessed the model's predictive accuracy. This was determined using R^2^ coefficients (Supplementary Table S2). In both plots, we found moderate R^2^ values for cases (0.48) and deaths (0.54), with interventions correlating with reduced COVID-19-related deaths and cases (except stay home requirements [p=0.50] and cancellation of public events [p=0.08]). Furthermore, the impact of each intervention on the predicted number of cases and deaths was analysed using coefficient plots (Figure 8). These plots displayed horizontal bars for each intervention, with the bar length representing the coefficient value. Positive coefficients indicated that increasing the intervention was associated with an increase in predicted cases/deaths. Conversely, negative coefficients suggested a decrease in predicted cases/deaths with an increase in the intervention. The absolute value of the coefficient reflected the strength of the effect, with larger values indicating a stronger impact on the prediction. Based on the coefficient plots, interventions such as public transport closures (coefficient value −166.56, p=0.01), workplace closures (coefficient value −150.06, p=0.01), contact tracing (coefficient value −74.85, p=0.01) and vaccination policy (coefficient value −47.06, p=0.04) had strong effects on reducing COVID-19 cases (Figure 8A). These intervention measures, except workplace closures (coefficient value 1.46, p=0.01) also correlated with a reduction in COVID-19-related deaths in the country (Figure 8B). Contact tracing (coefficient value −0.58, p=0.05) was also associated with a reduction in deaths, but the effect size was weaker, unlike public transport closures (coefficient value −2.43, p<0.001).

## Discussion

The global threat of the COVID-19 pandemic necessitated the adoption of NPIs to mitigate rapid spread of the SARS-CoV-2 virus. Despite the widespread implementation of NPIs, their long-term effectiveness is unknown. A robust evaluation framework for NPI effectiveness is required to guide future responses to emerging pathogens. This study used ML techniques to retrospectively evaluate the effect of NPIs on new cases and deaths in Nigeria during the COVID-19 pandemic. By leveraging clustering algorithms and regression analysis on temporal data, this study provided a preliminary assessment of NPI effectiveness.

The analysis of new COVID-19 cases showed short intervals between peaks of new cases during the early months of the pandemic (March–July 2020), suggesting rapid transmission and potential challenges in controlling the spread of the virus early on. A longer trough interval was observed subsequently from 27 January 2021 to 25 August 2021, indicating a period of decreased transmission due possibly to a successful introduction or stricter implementation of interventions to curb the spread of the virus. However, peak intervals narrowed shortly afterwards between 25 August 2021 and 29 December 2021, when the number of new cases reached a maximum peak before declining significantly. This demonstrated fluctuating transmission patterns that coincided with the relaxation of COVID-19 restrictions in Nigeria.^26^

Daily new COVID-19 deaths followed a somewhat different pattern compared with cases. The first reported COVID-19-linked death in the country occurred on 29 March 2020, with a peak observed on 6 May 2020, with approximately seven new (smoothed) deaths. Unlike the cases, there were multiple peaks of daily reported deaths within short intervals. This suggests a significant burden on healthcare systems, potential challenges in managing severe cases or possible lapses in NPI adherence and implementation. The maximum peak of new daily deaths was recorded on 8 September 2021. The last documented daily new death (smoothed) was reported on 18 September 2022, indicating a potential decline in mortality rates and the possibility of achieving control over the pandemic's impact on healthcare systems. The COVID-19 transmission and mortality patterns observed in the country aligned with trends observed in other African countries.^27^

Furthermore, when clustering algorithms were adopted to gain insights into the underlying patterns within intervention measures, the elbow plot initially suggested four clusters as optimal. However, silhouette analysis revealed that K=3 offered the highest score and met the criteria of having a score exceeding the average sample score. This highlights the importance of combining multiple metrics for robust K selection in K-means clustering, especially when the elbow method presents ambiguity.^28^ By reducing dimensionality, we effectively projected interventions onto a 2D plane,^29,30^ where closer proximity indicated greater similarity in policy characteristics and implementation effectiveness. Conversely, dispersed clusters represented interventions with diverse or potentially conflicting effects across measured features. This was exemplified by the distinct clustering of contact tracing measures.

Moreover, we integrated a linear regression model to pinpoint intervention effectiveness. While not exceptionally high, the model achieved somewhat moderate correlations based on the R^2^ values. This finding suggests the model had some success in predicting case and death outcomes per intervention. To determine which interventions had the most significant positive or negative coefficients (strongest effects) on predicted cases and deaths,^31,32^ we examined the coefficient analyses and observed significant associations between several NPIs and a reduction in predicted COVID-19 cases and deaths. For instance, public transport closures appeared to be highly impactful in reducing cases. Workplace closures, contact tracing and vaccination policy also displayed strong negative coefficients, implying their effectiveness in curbing case numbers. Public transport closures appeared to be particularly effective in reducing cases, potentially by limiting viral transmission across geographic areas. Workplace closures and robust contact tracing programs likely contributed by minimizing transmission within workplaces and communities. The impact of these interventions possibly translated to a decrease in predicted COVID-19 deaths as well.^33,34^ In addition, stay home requirements showed a significant association with reduced deaths. Contact tracing also correlated with a decline in deaths, although the effect size was weaker compared with other measures. These findings align with other studies that demonstrated the effectiveness of layered interventions over any single measure.^35,36^

However, it is important to note that correlation does not necessarily imply causation, as observed relationships between interventions and outcomes could be influenced by extraneous measures beyond the analysed NPIs.^37,38^ Furthermore, the analysis focused on a specific set of interventions that might not have been uniformly implemented at regional levels. In addition, we did not observe a clear elbow inflection point to identify the right K using the elbow method, which is a potential limitation to cluster determination. Moreover, the K-means algorithm adopted in this article is sensitive to the choice of centroids, and the optimal number of clusters could vary depending on the dataset. Future research could explore more robust K-means initialization techniques and consider incorporating other relevant features.

## Conclusions

This study presented a systematic ML approach for evaluating the efficacy of NPIs implemented during the COVID-19 pandemic. The findings theoretically offer decision support to policymakers and public health authorities by highlighting the relative effectiveness of various interventions to enhance pandemic response strategies and mitigation measures for future outbreaks.

## Supplementary Material

ihae065_Supplemental_Figures_and_Tables

## Data Availability

Data are available in a repository and can be accessed via 10.6084/m9.figshare.26982931. The Google Colab Python script used for data analysis and ML has been deposited in our GitHub page (https://github.com/oyebolakolapo/Retrospective-Evaluation-of-Non-Pharmaceutical-Interventions-against-COVID-19-in-Nigeria.git).

## References

[1] Baral S, Rao A, Rwema JOT et al. Competing health risks associated with the COVID-19 pandemic and early response: a scoping review. PLoS One. 2022;17(8):e0273389. 36037216 10.1371/journal.pone.0273389 PMC9423636

[2] Sempowski GD, Saunders KO, Acharya P et al. Pandemic preparedness: developing vaccines and therapeutic antibodies for COVID-19. Cell. 2020;181(7):1458–63. 32492407 10.1016/j.cell.2020.05.041 PMC7250787

[3] Zamir M, Shah Z, Nadeem F et al. Non pharmaceutical interventions for optimal control of COVID-19. Comput Methods Programs Biomed. 2020;196:105642. 32688137 10.1016/j.cmpb.2020.105642 PMC7339477

[4] Huang QM, Song WQ, Liang F et al. Non-pharmaceutical interventions implemented to control the COVID-19 were associated with reduction of influenza incidence. Front Public Health. 2022;10:773271. 35252083 10.3389/fpubh.2022.773271 PMC8894245

[5] Zhang Y, Quigley A, Wang Q et al. Non-pharmaceutical interventions during the roll out of covid-19 vaccines. BMJ. 2021;375:n2314. 34853011 10.1136/bmj.n2314 PMC8634371

[6] Gong M, Liu L, Wu C et al. Conducting clinical studies during the epidemics of communicable diseases: perspectives of methodology and health economics. Nan Fang Yi Ke Da Xue Xue Bao. 2020;40(3):353–7. 32376587 10.12122/j.issn.1673-4254.2020.03.11 PMC7167318

[7] Alfaqeeh M, Zakiyah N, Suwantika AA et al. Evaluation of global post-outbreak COVID-19 treatment interventions: a systematic review and bibliometric analysis of randomized controlled trials. J Multidiscip Healthc. 2023;16:4193–209. 38152831 10.2147/JMDH.S448786 PMC10752030

[8] Rid A, Emanuel EJ. Ethical considerations of experimental interventions in the Ebola outbreak. Lancet. 2014;384(9957):1896–9. 25155413 10.1016/S0140-6736(14)61315-5

[9] Ganegoda NC, Wijaya KP, Páez Chávez J et al. Reassessment of contact restrictions and testing campaigns against COVID-19 via spatio-temporal modeling. Nonlinear Dyn. 2022;107(3):3085–109. 34955605 10.1007/s11071-021-07111-w PMC8686823

[10] Iyaniwura SA, Rabiu M, David JF et al. Assessing the impact of adherence to non-pharmaceutical interventions and indirect transmission on the dynamics of COVID-19: a mathematical modelling study. Math Biosci Eng. 2021;18(6):8905–32. 34814328 10.3934/mbe.2021439

[11] Alfano V, Ercolano S. The efficacy of lockdown against COVID-19: a cross-country panel analysis. Appl Health Econ Health Policy. 2020;18(4):509–17. 32495067 10.1007/s40258-020-00596-3 PMC7268966

[12] Proverbio D, Kemp F, Magni S et al. Dynamical SPQEIR model assesses the effectiveness of non-pharmaceutical interventions against COVID-19 epidemic outbreaks. PLoS One. 2021;16(5):e0252019. 34019589 10.1371/journal.pone.0252019 PMC8139462

[13] Kermack WO, McKendrick AG. Contributions to the mathematical theory of epidemics—II. The problem of endemicity. Bull Math Biol. 1991;53(1–2):57–87. 2059742 10.1007/BF02464424

[14] Sun P, Li K. An SEIR model for assessment of current COVID-19 pandemic situation in the UK. medRxiv. 2020;doi: 10.1101/2020.04.12.20062588.

[15] Adiga A, Dubhashi D, Lewis B et al. Models for COVID-19 Pandemic: a comparative analysis. ArXiv. 2020;arXiv:2009.10014v1. 10.1007/s41745-020-00200-6 PMC7596173 33144763

[16] Cooper I, Mondal A, Antonopoulos CG. A SIR model assumption for the spread of COVID-19 in different communities. Chaos Solitons Fractals. 2020;139:110057. 32834610 10.1016/j.chaos.2020.110057 PMC7321055

[17] Fujiwara N, Onaga T, Wada T et al. Analytical estimation of maximum fraction of infected individuals with one-shot non-pharmaceutical intervention in a hybrid epidemic model. BMC Infect Dis. 2022;22:512. 35650534 10.1186/s12879-022-07403-5 PMC9157046

[18] Davies NG, Kucharski AJ, Eggo RM et al. Effects of non-pharmaceutical interventions on COVID-19 cases, deaths, and demand for hospital services in the UK: a modelling study. Lancet Public Health. 2020;5(7):e375–85. 32502389 10.1016/S2468-2667(20)30133-X PMC7266572

[19] Kucharski AJ, Klepac P, Conlan AJK et al. Effectiveness of isolation, testing, contact tracing, and physical distancing on reducing transmission of SARS-CoV-2 in different settings: a mathematical modelling study. Lancet Infect Dis. 2020;20(10):1151–60. 32559451 10.1016/S1473-3099(20)30457-6 PMC7511527

[20] Dairi A, Harrou F, Zeroual A et al. Comparative study of machine learning methods for COVID-19 transmission forecasting. J Biomed Inform. 2021;118:103791. 33915272 10.1016/j.jbi.2021.103791 PMC8074522

[21] Khan M, Mehran MT, Haq ZU et al. Applications of artificial intelligence in COVID-19 pandemic: a comprehensive review. Expert Syst Appl. 2021;185:115695. 34400854 10.1016/j.eswa.2021.115695 PMC8359727

[22] Matrajt L, Eaton J, Leung T et al. Optimizing vaccine allocation for COVID-19 vaccines: potential role of single-dose vaccination. medRxiv. 2021;doi: 10.1101/2020.12.31.20249099. PMC8187351 34103510

[23] Mathieu E, Ritchie H, Rodés-Guirao L et al. Coronavirus pandemic (COVID-19). OurWorldInData.org. 2020.

[24] Marutho D, Handaka SH, Wijaya E et al. The determination of cluster number at k-mean using elbow method and purity evaluation on Headline News. 2018 International Seminar on Application for Technology of Information and Communication, Semarang, Indonesia, 2018, pp. 533–8. doi: 10.1109/ISEMANTIC.2018.8549751.

[25] Rousseeuw PJ . Silhouettes: a graphical aid to the interpretation and validation of cluster analysis. J Comput Appl Math. 1987;20:53–65.

[26] Oyeleke S . FG lifts COVID-19 restrictions, midnight curfew. 6 April 2022. https://punchng.com/just-in-fg-lifts-covid-19-restrictions-midnight-curfew/

[27] Bwire G, Ario AR, Eyu P et al. The COVID-19 pandemic in the African continent. BMC Med. 2022;20:167. 35501853 10.1186/s12916-022-02367-4 PMC9059455

[28] Arbelaitz O, Gurrutxaga I, Muguerza J et al. An extensive comparative study of cluster validity indices. Pattern Recognit. 2013;46(1):243–56.

[29] Borg I, Groenen PJF. Modern multidimensional scaling: theory and applications, 2nd ed. New York: Springer Science + Business Media; 2005.

[30] Kang B, García García D, Lijffijt J et al. Conditional t-SNE: more informative t-SNE embeddings. Mach Learn. 2021;110(10):2905–40. 34840420 10.1007/s10994-020-05917-0 PMC8599264

[31] Rustagi V, Bajaj M, Singh TP et al. Analyzing the effect of vaccination over COVID cases and deaths in Asian countries using machine learning models. Front Cell Infect Microbiol. 2021;11:806265. 35223534 10.3389/fcimb.2021.806265 PMC8877421

[32] Almalki A, Gokaraju B, Acquaah Y et al. Regression analysis for COVID-19 infections and deaths based on food access and health issues. Healthcare (Basel). 2022;10(2):324. 35206938 10.3390/healthcare10020324 PMC8871757

[33] Moore S, Hill EM, Tildesley MJ et al. Vaccination and non-pharmaceutical interventions for COVID-19: a mathematical modelling study. Lancet Infect Dis. 2021;21(6):793–802. 33743847 10.1016/S1473-3099(21)00143-2 PMC7972312

[34] Ge Y, Zhang WB, Wu X et al. Untangling the changing impact of non-pharmaceutical interventions and vaccination on European COVID-19 trajectories. Nat Commun. 2022;13:3106. 35661759 10.1038/s41467-022-30897-1 PMC9166696

[35] Huluka DK, Ashagrie AW, Gebremariam TH et al. Strategic response to COVID-19 in Ethiopia. Public Health Action. 2022;12(4):191–4. 36561907 10.5588/pha.22.0007 PMC9716818

[36] Musoke D, Nalinya S, Lubega GB et al. The effects of COVID-19 lockdown measures on health and healthcare services in Uganda. PLoS Global Public Health. 2023;3(1):e0001494. 36963035 10.1371/journal.pgph.0001494 PMC10021763

[37] Gershman SJ, Ullman TD. Causal implicatures from correlational statements. PLoS One. 2023;18(5):e0286067. 37200364 10.1371/journal.pone.0286067 PMC10194916

[38] Gelman A, Stern H. The difference between “significant” and “not significant” is not itself statistically significant. Am Statistician. 2006;60(4):328–31.

