# Supplementary material for: Machine learning insights on the effectiveness of non-pharmaceutical interventions against COVID-19 in Nigeria
Source: Int Health. 2025 Jan 9;17(5):809–19. doi: 10.1093/inthealth/ihae065 (PMC12406770; doi:10.1093/inthealth/ihae065)

**Supplementary Table 1: Non-Pharmaceutical Interventions (NPI) and their categorization levels**

| **NPI** | **Category** | **Levels** |
| --- | --- | --- |
| Face Coverings | Policy Implementation | 0: No policy |
|  |  | 1: Recommended |
|  |  | 2: Required in specific shared/public spaces where social distancing is not feasible |
|  |  | 3: Required in all shared/public spaces where social distancing is not feasible |
|  |  | 4: Required outside the home at all times, irrespective of location or presence of others |
| School Closures | Policy Implementation | NaN: No data available |
|  |  | 0: No measures implemented |
|  |  | 1: Recommendations in place |
|  |  | 2: Required closures at certain levels |
|  |  | 3: Required closures at all levels |
| Workplace Closures | Policy Implementation | NaN: No data available |
|  |  | 0: No measures implemented |
|  |  | 1: Recommendations in place |
|  |  | 2: Required closures at certain levels |
|  |  | 3: Required closures at all levels |
| Public Event Cancellations | Policy Implementation | NaN: No data available |
|  |  | 0: No measures implemented |
|  |  | 1: Recommended cancellations |
|  |  | 2: Required cancellations |
| Stay-at-Home Policies | Policy Implementation | NaN: No measures implemented |
|  |  | 0: Recommended stay-at-home advisories |
|  |  | 1: Required stay-at-home orders with exceptions for essential activities (e.g., exercise, shopping, necessary trips) |
|  |  | 2: Required stay-at-home orders with minimal exceptions (e.g., leaving the house only once every few days) |
| Public Transport Closures | Policy Implementation | NaN: No data available |
|  |  | 0: No measures implemented |
|  |  | 1: Recommended closures or reduced service |
|  |  | 2: Required closures or significant restrictions on usage |
| Vaccination Policies | Availability Policy | 0: No availability |
|  |  | 1: Availability for one group (e.g., key workers, clinically vulnerable, elderly groups) |
|  |  | 2: Availability for two groups |
|  |  | 3: Availability for all three groups |
|  |  | 4: Availability for all three groups + partial additional availability for select broad groups or ages |
|  |  | 5: Universal availability |

**Supplementary Table 2: Linear regression model summary**


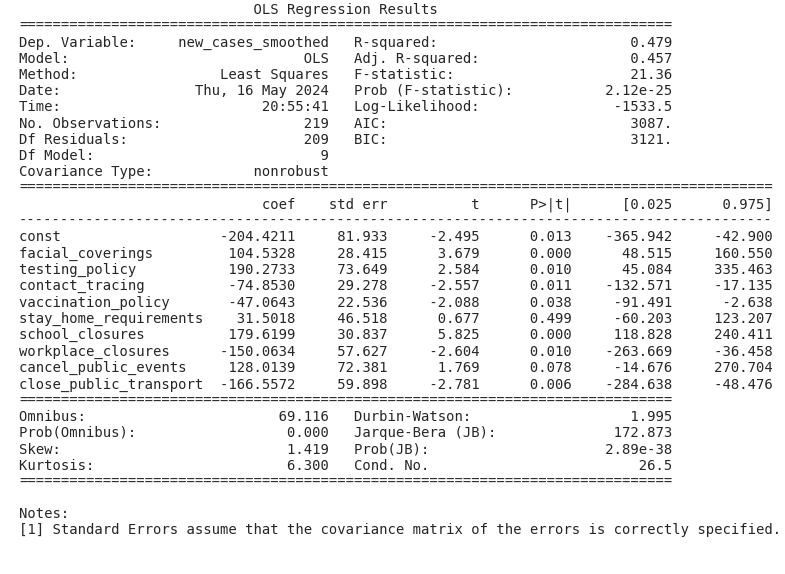


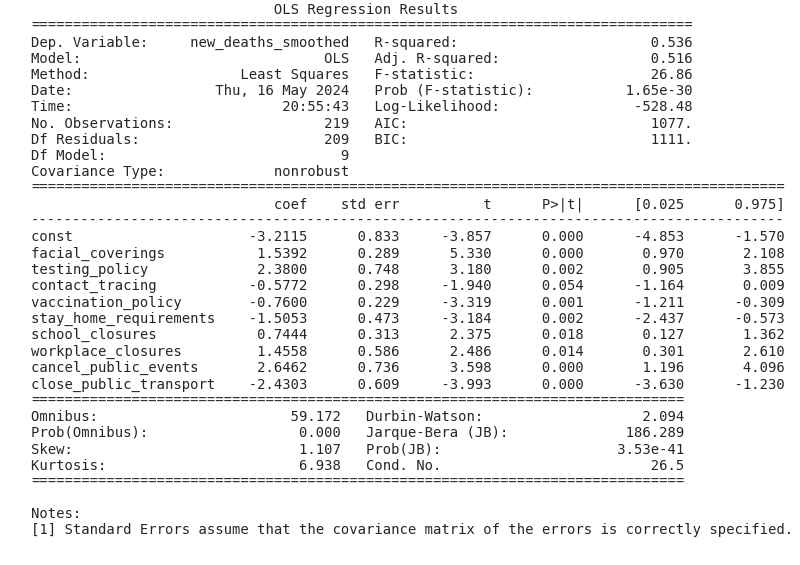

Supplement: ihae065_Supplemental_Figures_and_Tables [file ihae065_supplemental_figures_and_tables.zip › Supplementary Tables_revised.docx]
