# Supplementary figures and images for: Machine learning insights on the effectiveness of non-pharmaceutical interventions against COVID-19 in Nigeria
Source: Int Health. 2025 Jan 9;17(5):809–19. doi: 10.1093/inthealth/ihae065 (PMC12406770; doi:10.1093/inthealth/ihae065)

# Cleaned Data

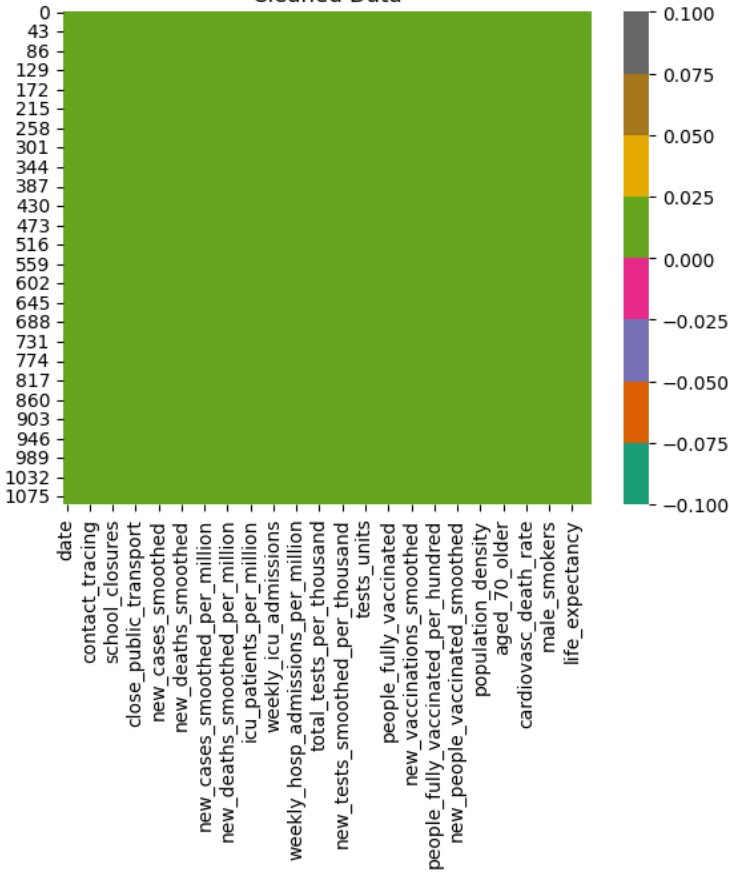

Supplement: ihae065_Supplemental_Figures_and_Tables [file ihae065_supplemental_figures_and_tables.zip › Supplementary Figure 1.pdf]

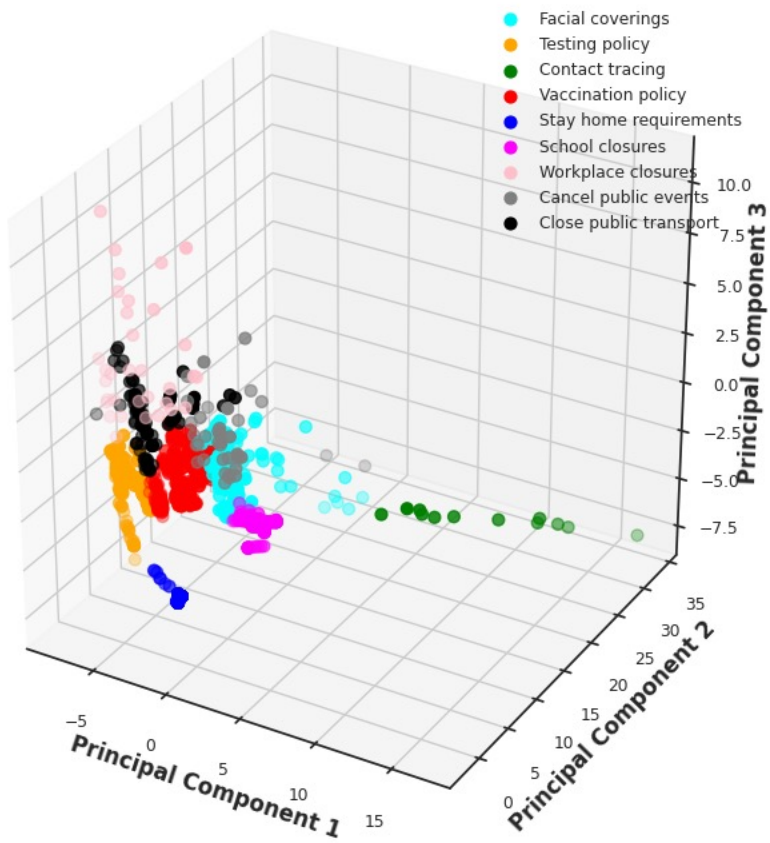

Supplement: ihae065_Supplemental_Figures_and_Tables [file ihae065_supplemental_figures_and_tables.zip › Supplementary Figure 2.pdf]
